# Supplementary material for: With a little help from my friends: importance of protist-protist interactions in structuring marine protistan communities in the San Pedro Channel
Source: mSystems. 2025 Jan 29;10(2):e01045-24. doi: 10.1128/msystems.01045-24 (PMC11834403; doi:10.1128/msystems.01045-24)
Supplement: Supplemental information — Supplemental figures and captions for supplemental tables. [file msystems.01045-24-s0001.docx]

**Supplementary Information**

**With a little help from my friends: Importance of protist-protist interactions in structuring marine protistan communities in the San Pedro Channel**

**Supplementary Figures**

**Figure S1:** Location of the San Pedro Ocean Time-series (SPOT) station (33°33′N, 118°24′W).

**Figure S2:** Nonmetric multidimensional scaling (NMDS) analysis comparing ASV-level microbial eukaryote diversity at the surface and DCM. Ellipses represent 95% confidence intervals.

**Figure S3:** Changes in community similarity plotted as a function of changes in ecosystem parameters. A principal component analysis was carried out using the environmental and biological ecosystem properties that were measured throughout the SPOT time-series (Table S2). The first principal component (PC1; that explained the largest fraction of the variability between samples) was extracted. The Bray-Curtis similarity for every pair of samples in the SPOT time-series dataset was plotted as a function of the Euclidean distance of the PC1 scores. The best-fit lines for the data are projected on the plots in red.

**Figure S4:** Subnetworks depicting the PIDA-supported parasitic relationships identified in the surface (a, b) and DCM (c, d) network outputs. Interactions involving Syndiniales parasites were considered PIDA-supported when the Syndiniales group and host genus in the Protist Interaction DAtabase (PIDA; ref. 13) matched a network prediction. All other parasitic interactions that were considered PIDA-supported matched a parasitic relationship documented in the PIDA database at the genus level. Panels a and c show putative relationships involving Group-I Syndiniales parasites and *Cryothecomonas* parasites, while panels b and d show putative relationships involving Group-II Syndiniales parasites. Blue lines depict relationships that were found in both the surface and DCM network outputs. Circles represent individual ASVs that were involved in network-predicted associations and the circle colors delineate the major taxonomic affiliations of the ASVs. Letters denote the genus-level taxonomic identification for all ASVs that are not a Syndiniales parasite or a dinoflagellate (A=*Eutintinnus*, B=*Pelagostrobilidium*, C=*Strombidium*, D=*Cryothecomonas*, E=*Chaetoceros*, F=*Rhizosolenia*, G=*Thalassiosira*). The full taxonomic information for each ASV in these PIDA-supported parasitic subnetworks is documented in Table S4.

**Figure S5:** Subnetworks depicting the PIDA-supported predator-prey and kleptoplastic relationships identified in the surface (a) and DCM (b) network outputs. Network predictions that matched a predator-prey or kleptoplastic relationship recorded in the Protist Interaction DAtabase (PIDA; ref. 13) at the genus level were considered PIDA-supported. Blue lines depict relationships that were found in both the surface and DCM network outputs. Circles represent individual ASVs that were involved in network-predicted associations and the circle colors delineate the major taxonomic affiliations of the ASVs. Letters denote the genus-level taxonomic identification for all ASVs (A=*Pyramimonas,* B=*Teleaulax,* C=*Chrysochromulina*, D=*Phaeocystis,* E=*Pelagostrobilidium*, F=*Strombidium,* G=*Alexandrium,* H=*Dinophysis*, , I=*Gonyaulax*, J=*Gymnodinium*, K=*Gyrodinium*, L=*Heterocapsa*, M=*Karlodinium*, N=*Lingulodinium*, O=*Luciella*, P=*Prorocentrum*, Q=*Protoperidinium*, R=Scrippsiella, S=*Chaetoceros*, T=*Nitzschia,* U=*Thalassiosira*). The full taxonomic information for each ASV in these PIDA-supported subnetworks is documented in Table S5.

**Figure S6:** Putative, novel ASV-ASV interactions plotted over time. The ASV abundance data were CLR-transformed, and the spearman correlation coefficient (SCC) was calculated for pairs of ASVs that were involved in a network-predicted interaction. Some of the strongest network-predicted interactions at the surface (a-h) and DCM (i-p) that were not found in the PIDA database were visualized over the 14-year and nine-month time-series. The full taxonomic information for each ASV involved in these putative, novel interactions is documented in Table S7.

**Supplementary Table Legends**

*Tables can be found in TableS1_S7.xlsx*

**Table S1:** Date and depth of sample collection for the 18S-V4 rRNA gene amplicon data that were used in the analyses carried out in this study.

**Table S2:** Environmental and biological parameters that were used in the analyses carried out in this study.

**Table S3:** Percent of variance in protistan community composition explained by each of the redundancy analysis (RDA) axes for the surface and DCM communities.

**Table S4:** Full list of the PIDA-supported parasitic relationships that were detected in the surface and DCM SPOT networks. The spearman correlation coefficient (SCC) and adjusted *p*-value for each interaction is reported. P-value adjustments were carried out using the holm adjustment method.

**Table S5:** Full list of the PIDA-supported predator-prey and kleptoplastic relationships that were detected in the surface and DCM SPOT networks. The spearman correlation coefficient (SCC) and adjusted *p*-value for each interaction is reported. P-value adjustments were carried out using the holm adjustment method.

**Table S6:** Full list of the other PIDA-supported symbiotic relationships that were detected in the surface and DCM SPOT networks. The spearman correlation coefficient (SCC) and adjusted *p*-value for each interaction is reported. P-value adjustments were carried out using the holm adjustment method.

**Table S7:** Taxonomic information, correlation coefficients, and adjusted *p*-values for all of the putative, novel ASV-ASV interactions visualized in Figure 7 and Figure S7. The *p*-values were adjusted using the holm adjustment method.
